# Clinical laboratory characteristics and gene mutation spectrum of *Ph*‐negative MPN patients with atypical variants of *JAK2*, *MPL*, or *CALR*

**DOI:** 10.1002/cam4.7123

**Published:** 2024-04-15

**Authors:** Zhanlong Wang, Xin Tian, Jinyu Ma, Yuhui Zhang, Wenru Ta, Yifan Duan, Fengli Li, Hong Zhang, Long Chen, Shaobin Yang, Enbin Liu, Yani Lin, Weiping Yuan, Kun Ru, Jie Bai

**Affiliations:** ^1^ Department of Hematology The Second Hospital of Tianjin Medical University Tianjin China; ^2^ Sino‐US Diagnostics Lab Tianjin Enterprise Key Laboratory of AI‐aided Hematopathology Diagnosis Tianjin China; ^3^ State Key Laboratory of Experimental Hematology, Institute of Hematology and Blood Disease Hospital Chinese Academy of Medical Sciences and Peking Union Medical College Tianjin China; ^4^ Department of Pathology and Lab Medicine Shandong Cancer Hospital Jinan China

**Keywords:** atypical variant, classical mutation, co‐existence, driver genes, myeloproliferative neoplasms

## Abstract

**Objective:**

To evaluate the incidence, clinical laboratory characteristics, and gene mutation spectrum of *Ph*‐negative MPN patients with atypical variants of *JAK2*, *MPL*, or *CALR*.

**Methods:**

We collected a total of 359 *Ph*‐negative MPN patients with classical mutations in driver genes *JAK2*, *MPL*, or *CALR*, and divided them into two groups based on whether they had additional atypical variants of driver genes *JAK2*, *MPL*, or *CALR*: 304 patients without atypical variants of driver genes and 55 patients with atypical variants of driver genes. We analyzed the relevant characteristics of these patients.

**Results:**

This study included 359 patients with *Ph*‐negative MPNs with *JAK2*, *MPL*, or *CALR* classical mutations and found that 55 (15%) patients had atypical variants of *JAK2*, *MPL*, or *CALR*. Among them, 28 cases (51%) were male, and 27 (49%) were female, with a median age of 64 years (range, 21–83). The age of ET patients with atypical variants was higher than that of ET patients without atypical variants [70 (28–80) vs. 61 (19–82), *p* = 0.03]. The incidence of classical *MPL* mutations in ET patients with atypical variants was higher than in ET patients without atypical variants [13.3% (2/15) vs. 0% (0/95), *p* = 0.02]. The number of gene mutations in patients with atypical variants of driver genes PV, ET, and Overt‐PMF is more than in patients without atypical variants of PV, ET, and Overt‐PMF [PV: 3 (2–6) vs. 2 (1–7), *p* < 0.001; ET: 4 (2–8) vs. 2 (1–7), *p* < 0.05; Overt‐PMF: 5 (2–9) vs. 3 (1–8), *p* < 0.001]. The incidence of *SH2B3* and *ASXL1* mutations were higher in MPN patients with atypical variants than in those without atypical variants (*SH2B3*: 16% vs. 6%, *p* < 0.01; *ASXL1*: 24% vs. 13%, *p* < 0.05).

**Conclusion:**

These data indicate that classical mutations of *JAK2*, *MPL*, and *CALR* may not be completely mutually exclusive with atypical variants of *JAK2*, *MPL*, and *CALR*. In this study, 30 different atypical variants of *JAK2*, *MPL*, and *CALR* were identified, *JAK2 G127D* being the most common (42%, 23/55). Interestingly, *JAK2 G127D* only co‐occurred with *JAK2* ^*V617F*^ mutation. The incidence of atypical variants of *JAK2* in *Ph*‐negative MPNs was much higher than that of the atypical variants of *MPL* and *CALR*. The significance of these atypical variants will be further studied in the future.

## Graphical abstract

The driver genes *JAK2*, *MPL*, and *CALR* may not be completely mutually exclusive for classical mutations and atypical variants. There is a co‐existence correlation between *JAK2^V617F^* and *JAK2 G127D*. *Ph*‐negative MPN patients with atypical variants of driver genes have a higher mutation rate of *SH2B3* and *ASXL1*, which may indicate poor prognosis.

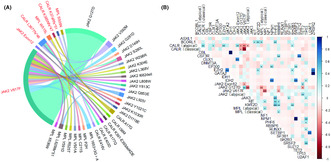

## 1 INTRODUCTION

Classical *Ph*‐negative myeloproliferative neoplasms (MPNs) are a type of clonal hematopoietic stem cell (HSC) disease. According to the latest WHO classification of hematopoietic and lymphoid tissues, mainly include three classical subtypes: polycythaemia vera (PV), essential thrombocythaemia (ET), and primary myelofibrosis (PMF). These diseases often involve abnormal effective proliferation of one or more lineages of granulocytes, erythrocytes, and megakaryocytes, which can lead to an increase in neutrophils, red blood cells, and platelets.^1^, ^2^, ^3^ The primary pathogenic mechanism of *Ph*‐negative MPN is the persistent activation of the JAK–STAT signaling pathway caused by driver gene mutations in *JAK2* ^*V617F*^, *JAK2 Exon12*, *MPL W515L/K/R/A/G/S*, *MPL S505N*, and *CALR Exon 9 ins/del*.^2^, ^4^, ^5^, ^6^, ^7^, ^8^ The classical mutation of driver genes positive is one of the main diagnostic criteria for *Ph*‐negative MPN. The epigenetic gene mutations such as *TET2*, *ASXL1*, *DNMT3A*, and *EZH2* are also closely associated with disease onset and progression, increasing numbers of mutant genes and increased mutant burden (drivers and non‐drivers) during disease clonal evolution can also lead to disease progression.^9^, ^10^, ^11^, ^12^

Classical mutations of driver genes *JAK2*, *MPL*, and *CALR* in *Ph*‐negative MPN are considered to be mutually exclusive.^13^, ^14^ With the development of sequencing technology, not only have there been cases of co‐existence of classical mutations of *JAK2*, *MPL*, and *CALR*, but also cases of co‐existence of classical mutations and atypical variants of *JAK2*, *MPL*, and *CALR* have been discovered.^7^, ^15^, ^16^, ^17^, ^18^, ^19^, ^20^ Atypical variants of driver genes have been researched more in triple‐negative MPN and familial MPN, some of which, such as *JAK2 V625F*, *JAK2 F556V*, *MPL S204P*, *MPL Y591N/D*, *MPL A497‐L498ins4*, *MPL W515RQ516E*, etc., have been proven to be gain‐of‐function mutations, which can lead to different degrees of activation in the JAK–STAT signaling pathway.^21^, ^22^, ^23^ At present, there is scant research on the *Ph*‐negative MPN cohort where classical mutations and atypical variants of driver genes coexist. There has been no previous study on the gene mutation spectrum of *Ph*‐negative MPN patients in which classical mutations and atypical variants of driver genes coexist. For the first time, we used high sensitivity next generation sequencing technology to study Chinese *Ph*‐negative MPN patients, filling a gap in the field.

In this study, our target population was *Ph*‐negative MPN patients with atypical variants of driver genes. We studied the co‐existence of classical mutations and atypical variants in driver genes *JAK2*, *MPL*, and *CALR*, and explore the incidence, gene mutation spectrum, and clinical laboratory characteristics of *Ph*‐negative MPN patients who are positive for classical mutations in driver genes and also have additional atypical variants of *JAK2*, *MPL*, or *CALR*.

## 2 MATERIALS AND METHODS

### 2.1 Study population

We collected a total of 359 cases diagnosed *Ph*‐negative MPN with classical mutations of driver genes positive from August 2017 to October 2022. All were cases of initial diagnosis or first referral. The MPN diagnosis was in accordance with the 2017 version of the WHO classification of hematopoietic and lymphoid tumors.^24^ According to whether there were additional atypical variants of the driver genes, *Ph*‐negative MPN patients were divided into two groups: (1) There were 304 patients without atypical variants of driver genes [PV, *n* = 115; ET, *n* = 95; and PMF, *n* = 94 (including 33 cases of Pre‐PMF and 61 cases of Overt‐PMF)]. (2) There were 55 patients with atypical variants of driver genes [PV, *n* = 19; ET, *n* = 15; and PMF, *n* = 21 (including 6 cases of Pre‐PMF and 15 cases of Overt‐PMF)]. A focused analysis was conducted on the incidence, clinical laboratory characteristics and next generation sequencing results of 55 *Ph*‐negative MPN patients, who were positive for classical mutations of *JAK2*, *MPL*, or *CALR* and also had atypical variants of *JAK2*, *MPL*, or *CALR*.

### 2.2 Bone marrow pathological diagnosis analysis

Bone marrow trephine biopsy specimens are prepared for paraffin embedding, using H&E automatic stainer (Coverstainer, DAKO) for Hematoxylin and Eosin (HE) staining, manual method for reticular fiber and periodic acid schiff (BASO, China) staining. According to the 2017 WHO Classification of Tumors of Hematopoietic and Lymphoid Tissues,^24^ OLYMPUS BX53 biological microscope is used for disease classification diagnosis and reticular fibrosis level assessment of *Ph*‐negative MPN.

### 2.3 Chromosomal karyotype analysis

Bone marrow specimens were treated with RPMI1640 culture medium (YUANDE, China), and after mitosis of nucleated cells, colchicine (0.4 μg/mL) was used to arrest cells in the metaphase. The cells were then treated with a hypotonic solution (0.4% KCl), and a fixing solution (methanol:glacial acetic acid = 3:1) was used to fix the cells. The cells were stained for R or G bands with Giemsa stain solution (10% Giemsa staining solution) after spreading on slides, and completed bands were scanned for chromosomes in metaphase using an automatic chromosome scanning device (M9120, BEION, China). At least 20 metaphases should be analyzed for chromosome inspection whenever possible. The results of karyotyping were described according to the 2020 International System for Human Cytogenetic Nomenclature (ISCN).

### 2.4 Molecular analysis

Bone marrow aspirate specimen in EDTA anticoagulant, separate individual nucleated cells from the bone marrow aspirate specimen using density gradient centrifugation, and extract DNA using the Blood Genome DNA Extraction Kit (TIANGEN, China). The sequencing of 42 hematological tumor related genes in 359 *Ph*‐negative MPN patients was performed using the Illumina NextSeq 550 high‐throughput sequencing platform. Regions of the sequencing include the entire exon regions from the 42 genes. The minimum variant allele frequency (VAF) to be 1%. The average sequencing depth was ≥1000×. Mutant genes with mutation rates of 40%–60% are used to verify the germline origin of hair follicles using Sanger sequencing. Data were filtered and screened using public databases such as dbSNP, 1000Genomes, gnomAD, COSMIC, ClinVar, and prediction software such as Polyphen2, SIFT, and Mutation Taster. The 42 hematological tumor related genes included *ABL1*, *ASXL1*, *BCORL1*, *CALR*, *CBL*, *CSF3R*, *CUX1*, *DDX41*, *DNMT3A*, *EP300*, *ETNK1*, *ETV6*, *EZH2*, *FLT3*, *GATA2*, *IDH1*, *IDH2*, *IKZF1*, *JAK2*, *JAK3*, *KIT*, *KMT2D*, *KRAS*, *MPL*, *NF1*, *NPM1*, *NRAS*, *PAX5*, *PHF6*, *PTPN11*, *RBBP6*, *RUNX1*, *SETBP1*, *SF3B1*, *SH2B3*, *SRSF2*, *SUZ12*, *TET2*, *TP53*, *U2AF1*, *WT1*, *and ZRSR2*.

### 2.5 Statistical analysis

All data were statistically analyzed using IBM SPSS 26.0 and R software (Version 4.2.2). For intergroup comparisons of quantitative data that conform to a normal distribution, single‐factor analysis of variance or *t*‐tests were used, and the results were expressed as means ± standard deviations. Non‐parametric tests such as the Mann–Whitney *U*‐test were used for non‐normally distributed data, and the results were expressed as medians (ranges). For categorical data, chi‐squared tests (*n* ≥ 40 and *T* ≥ 5) or Fisher's exact probability tests (*n* ≥ 40 and 1 ≤ *T* < 5; *n* < 40 or *T* < 1) were used for intergroup comparisons. Differences were considered statistically significant when *p* < 0.05.

## 3 RESULTS

### 3.1 Patient cohorts and characteristics

Among the 359 *Ph*‐negative MPN patients with positive classical mutations in driver genes, there were 304 patients without atypical variants of driver genes [PV, *n* = 115; ET, *n* = 95; and PMF, *n* = 94 (including 33 cases of Pre‐PMF and 61 cases of Overt‐PMF)], and 55 patients with atypical variants [PV, *n* = 19; ET, *n* = 15; and PMF, *n* = 21 (including 6 cases of Pre‐PMF and 15 cases of Overt‐PMF)]. The clinical laboratory characteristics of these two types of *Ph*‐negative MPN patients are shown in Table 1. Patients with atypical variants of driver genes accounted for 15% (55/359) of the total *Ph*‐negative MPN, including 14% (19/134) in PV, 14% (15/110) in ET, and 18% (21/115) in PMF. Among the 55 patients with atypical variants of driver genes, there were 28 males and 27 females, with a median age of 64 (range: 21–83) years old. The age of ET patients with atypical variants of driver genes was higher than ET patients without atypical variants [70 (28–80) vs. 61 (19–82), *p* = 0.03], the age of PV, Pre‐PMF and Overt‐PMF patients with atypical variants of driver genes was no significant difference than PV, Pre‐PMF and Overt‐PMF patients without atypical variants. The incidence of classical *MPL* mutations in ET patients with atypical variants of driver genes was higher than ET patients without atypical variants [13.3% (2/15) vs. 0% (0/95), *p* = 0.02], the incidence of other classical mutated driver genes was not significantly different between the *Ph*‐negative MPN subgroup with atypical variants of driver genes and the subgroup without atypical variants. The number of gene mutations in patients with atypical variants of driver genes PV, ET, and Overt‐PMF is more than in patients without atypical variants of PV, ET, and Overt‐PMF [PV: 3 (2–6) vs. 2 (1–7), *p* < 0.001; ET: 4 (2–8) vs. 2 (1–7), *p* < 0.05; Overt‐PMF: 5 (2–9) vs. 3 (1–8), *p* < 0.001].

**TABLE 1:** Clinical and laboratory characteristics of 55 patients with atypical variants of driver genes MPN and 304 patients without atypical variants of driver genes MPN.

	Total (*N* = 359)	MPN with atypical variants of driver genes (*N* = 55)				MPN without atypical variants of driver genes (*N* = 304)				*p*‐value			
		(A) PV (*n* = 19)	(B) ET (*n* = 15)	(C) Pre‐PMF (*n* = 6)	(D) Overt‐PMF (*n* = 15)	(E) PV (*n* = 115)	(F) ET (*n* = 95)	(G) Pre‐PMF (*n* = 33)	(H) Overt‐PMF (*n* = 61)	A versus E	B versus F	C versus G	D versus H
Male:female	171/188	10/9	8/7	3/3	7/8	59/56	38/57	18/15	28/33	0.92	0.33	1.00	0.96
Media age (range)	63 (19–92)	61 (21–74)	70 (28–80)	60 (46–71)	69 (55–83)	63 (21–83)	61 (19–82)	64 (22–91)	66 (21–92)	0.30	**0.03** ^*^	0.36	0.15
WBC (×10^9^/L), median (range)	11.9 (1.8–171.0)	12.5 (5.5–26.9)	9.3 (3.8–24.3)	9.6 (7.3–16.9)	14.5 (2.6–123.0)	14.2 (5.3–171.0)	9.9 (4.6–28.0)	10.0 (3.7–46.9)	12.4 (1.8–56.5)	0.47	0.95	0.83	0.51
Hb (g/L, median (range)	150 (40–242)	184.5 (160–242)	135 (77–163)	124 (103–160)	118 (70–154)	187 (160–238)	139 (87–166)	134 (57–165)	129 (40–164)	0.74	0.75	0.88	0.47
PLT (×10^9^/L), median (range)	700 (23–2705)	518 (173–1224)	732 (467–1261)	1003 (357–2227)	748 (172–1835)	529 (161–1544)	810 (350–2570)	892 (217–2705)	701 (23–2200)	0.84	0.25	0.57	0.88
Karotype (abnormal), *n* (%)	22/285 (7.7%)	1/17 (5.9%)	2/13 (15.4%)	0/5 (0%)	1/10 (10%)	8/98 (8.2%)	2/75 (2.7%)	2/24 (8.3%)	6/43 (14.0%)	1.00	0.10	1.00	1.00
Number of gene mutations, median (range)	3 (1–9)	3 (2–6)	4 (2–8)	4 (3–7)	5 (2–9)	2 (1–7)	2 (1–7)	3 (1–9)	3 (1–8)	**<0.001** ^***^	**<0.05** ^*^	0.09	**<0.001** ^***^
MF ≥ 2, *n* (%)	76/359 (21.2%)	0/19 (0%)	0/15 (0%)	0/6 (0%)	15/15 (100%)	0/115 (0%)	0/95 (0%)	0/33 (0%)	61/61 (100%)				
Driver gene classical mutation, *n* (%)										*p*‐value			
										OR (95% CI)			
										A versus E	B versus F	C versus G	D versus H
*JAK2* ^*V617F*^	288/359 (80.2%)	18/19 (94.7%)	9/15 (60%)	5/6 (83.3%)	13/15 (86.7%)	113/115 (98.3%)	71/95 (74.7%)	21/33 (63.6%)	38/61 (62.3%)	*p* = 0.37	*p* = 0.35	*p* = 0.64	*p* = 0.12
										OR = 3.14 (0.27–36.43)	OR = 1.98 (0.64–6.12)	OR = 0.35 (0.04–3.36)	OR = 0.25 (0.05–1.23)
*JAK2 Exon12*	3/359 (0.8%)	1/19 (5.3%)	0/15 (0%)	0/6 (0%)	0/15 (0%)	2/115 (1.7%)	0/95 (0%)	0/33 (0%)	0/61 (0%)	*p* = 0.37	*p* = 1.00	*p* = 1.00	*p* = 1.00
										OR = 0.32 (0.03–3.70)	OR = 6.16 (0.12–322.08)	OR = 5.15 (0.09–283.91)	OR = 0.55 (0.06–4.86)
*MPL Exon10*	12/359 (3.3%)	0/19 (0%)	2/15 (13.3%)	0/6 (0%)	1/15 (6.7%)	0/115 (0%)	0/95 (0%)	2/33 (6.1%)	7/61 (11.5%)	*p* = 1.00	***p* = 0.02** ^*^	*p* = 1.00	*p* = 1.00
										OR = 5.92 (0.11–307.37)	OR = 35.37 (1.61–776.86)	OR = 0.97 (0.04–22.65)	OR = 1.82 (0.21–15.99)
*CALR Exon9*	56/359 (15.6%)	0/19 (0%)	4/15 (26.7%)	1/6 (16.7%)	1/15 (6.7%)	0/115 (0%)	24/95 (25.3%)	10/33 (30.3%)	16/61 (26.2%)	*p* = 1.00	*p* = 1.00	*p* = 0.66	*p* = 0.17
										OR = 5.92 (0.11–307.37)	OR = 0.93 (0.27–3.19)	OR = 2.17 (0.22–21.08)	OR = 4.98 (0.61–40.95)

Abbreviations: ET, essential thrombocythemia; Hb, hemoglobin; PLT, platelet; PMF, primary myelofibrosis; PV, polycythemia vera; WBC, white blood cell.

Bold represents *p*‐values with statistical differences, *p < 0.05, **p < 0.01, ***p < 0.001.

**FIGURE 1:**
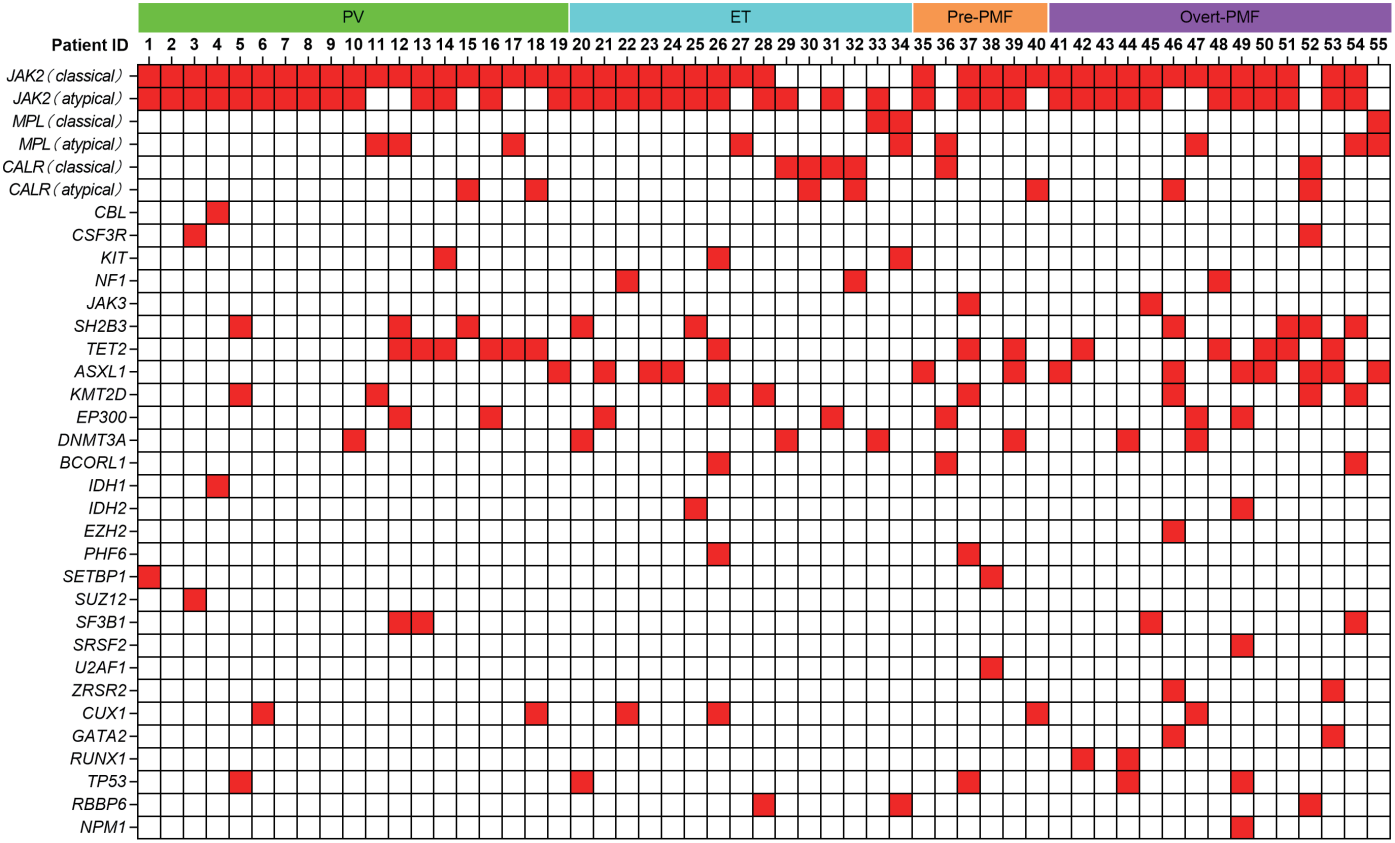
The gene mutation spectrum of 55 *Ph*‐negative MPN patients with atypical variants of the driver genes. Patients 1–19 (PV), patients 20–34 (ET), patients 35–40 (Pre‐PMF), patients 41–55 (Overt‐PMF).

Among the 359 *Ph*‐negative MPN patients, 16% (45/288) of the 288 patients *JAK2* ^*V617F*^ positive had atypical variants of *JAK2*, *MPL*, or *CALR*; 33% (1/3) of the 3 patients *JAK2 Exon12* mutation positive had atypical variants of *CALR*; 25% (3/12) of the 12 patients *MPL Exon10* classical mutation positive had atypical variants of *JAK2* or *MPL*; and 11% (6/56) of the 56 patients *CALR Exon9* classical mutation positive had atypical variants of *JAK2*, *MPL*, or *CALR*. Atypical variants of *JAK2*, *MPL*, and *CALR* can occur in any type of *Ph*‐negative MPN (PV, ET and PMF) (Figure 1).

### 3.2 Gene mutation spectrum analysis

Out of 55 *Ph*‐negative MPN patients with classical mutations and atypical variants in the driver genes, 53 had a classical mutation with an atypical variant (96%, 53/55), and 2 had a classical mutation with two atypical variants (4%, 2/55) (Table S1; Figure S1). The occurrence rates of driver genes classical mutations were *JAK2* (84%), *CALR* (11%), *MPL* (5%). The occurrence rates of driver genes atypical variants were *JAK2* (73%), *CALR* (13%), *MPL* (16%). Among the classical mutations of the driver genes in 55 patients, 18 were *JAK2* ^*V617F*^ and 1 was *JAK2 Exon12* in PV; 9 were *JAK2* ^*V617F*^, 4 were *CALR exon9* and 2 were *MPL W515L* in ET; 5 were *JAK2* ^*V617F*^ and 1 was *CALR Exon9* in Pre‐PMF; in Overt‐PMF, 13 were *JAK2* ^*V617F*^, 1 was *CALR Exon9* and 1 was *MPL S505N* (Figure S1). There is a co‐existence relationship between *JAK2* classical mutations and *JAK2* atypical variants (*p* < 0.01), *CALR* classical mutations and *CALR* atypical variants (*p* < 0.01), *MPL* classical mutations and *MPL* atypical variants (*p* < 0.05) (Figure S2). The frequency of non‐driver gene mutations, in descending order, was *TET2* (26%), *ASXL1* (24%), *SH2B3* (16%), *KMT2D* (15%), *EP300* and *DNMT3A* (13%), *CUX1* (11%), *TP53* (9%), *SF3B1* (7%), *BCORL1*, *KIT*, *NF1* and *RBBP6* (5%), *CSF3R*, *GATA2*, *IDH2*, *JAK3*, *PHF6*, *RUNX1*, *SETBP1* and *ZRSR2* (4%), *CBL*, *EZH2*, *IDH1*, *NPM1*, *SRSF2*, *SUZ12* and *U2AF1* (2%).

In 304 *Ph*‐negative MPN patients without atypical variants of driver genes, the occurrence rates of classical mutations in driver genes were *JAK2* (81%), *CALR* (16%), *MPL* (3%). The frequency of non‐driver gene mutations, in descending order, was *TET2* (23%), *KMT2D* (14%), *ASXL1* (13%), *DNMT3A* (12%), *CUX1* and *EP300* (9%), *RBBP6* (7%), *BCORL1* and *SH2B3* (6%), *CBL*, *SETBP1* and *CSF3R* (5%), *SRSF2*, *ABL1*, *SF3B1* and *KIT* (4%), *TP53*, *ZRSR2*, *NF1*, *IDH1*and *RUNX1* (3%), *EZH2*, *IDH2*, and *FLT3* (2%), *ETNK1*, *JAK3*, *WT1*, *ETV6*, *NPM1*, *PTPN11*, *U2AF1*, *DDX41*, *PAX5*, *PHF6*, *SUZ12* (1%).

All non‐driver mutant genes in 359 *Ph*‐negative MPN patients are categorized according to the function of the gene pathway, and the incidence of mutations is in descending order: chromatin modification genes, DNA methylation genes, signal transduction genes, transcriptional factor genes, RNA splicing genes, tumor suppressor genes and others (Figure S3). Analyze the occurrence rate of non‐driver gene mutations that are simultaneously >5% in the group MPN with atypical variants of driver genes and the group MPN without atypical variants of driver genes, the incidence of *SH2B3* and *ASXL1* mutations was higher in the MPN with atypical variants of driver genes than in the MPN without atypical variants of driver genes (*SH2B3*: 16% vs. 6%, *p* < 0.01; *ASXL1*: 24% vs. 13%, *p* < 0.05), there were no significant differences between the other mutated genes (Table 2).

**TABLE 2:** Compare the occurrence rate of non‐driver gene mutations that are simultaneously >5% in the group MPN with atypical variants of driver genes and the group MPN without atypical variants of driver genes.

Mutation genes	Total *n* (%)	MPN with atypical variants of driver genes *n* (%)	MPN without atypical variants of driver genes *n* (%)	*p‐*value
*TET2*	85/359 (23.7%)	14/55 (25.5%)	71/304 (23.4%)	0.736
*ASXL1*	53/359 (14.8%)	13/55 (23.6%)	40/304 (13.2%)	0.044*
*SH2B3*	26/359 (7.2%)	9/55 (16.4%)	17/304 (5.6%)	0.009**
*KMT2D*	51/359 (14.2%)	8/55 (14.5%)	43/304 (14.1%)	0.938
*EP300*	33/359 (9.2%)	7/55 (12.7%)	26/304 (8.6%)	0.324
*CUX1*	33/359 (9.2%)	6/55 (10.9%)	27/304 (8.9%)	0.632
*DNMT3A*	42/359 (11.7%)	7/55 (12.7%)	35/304 (11.5%)	0.797
*BCORL1*	20/359 (5.6%)	3/55 (5.5%)	17/304 (5.6%)	1.000
*RBBP6*	24/359 (6.7%)	3/55 (5.5%)	21/304 (6.9%)	1.000

* *p* < 0.05.

** *p* < 0.01.

### 3.3 *JAK2* , *MPL* , and *CALR* atypical variants

**FIGURE 2:**
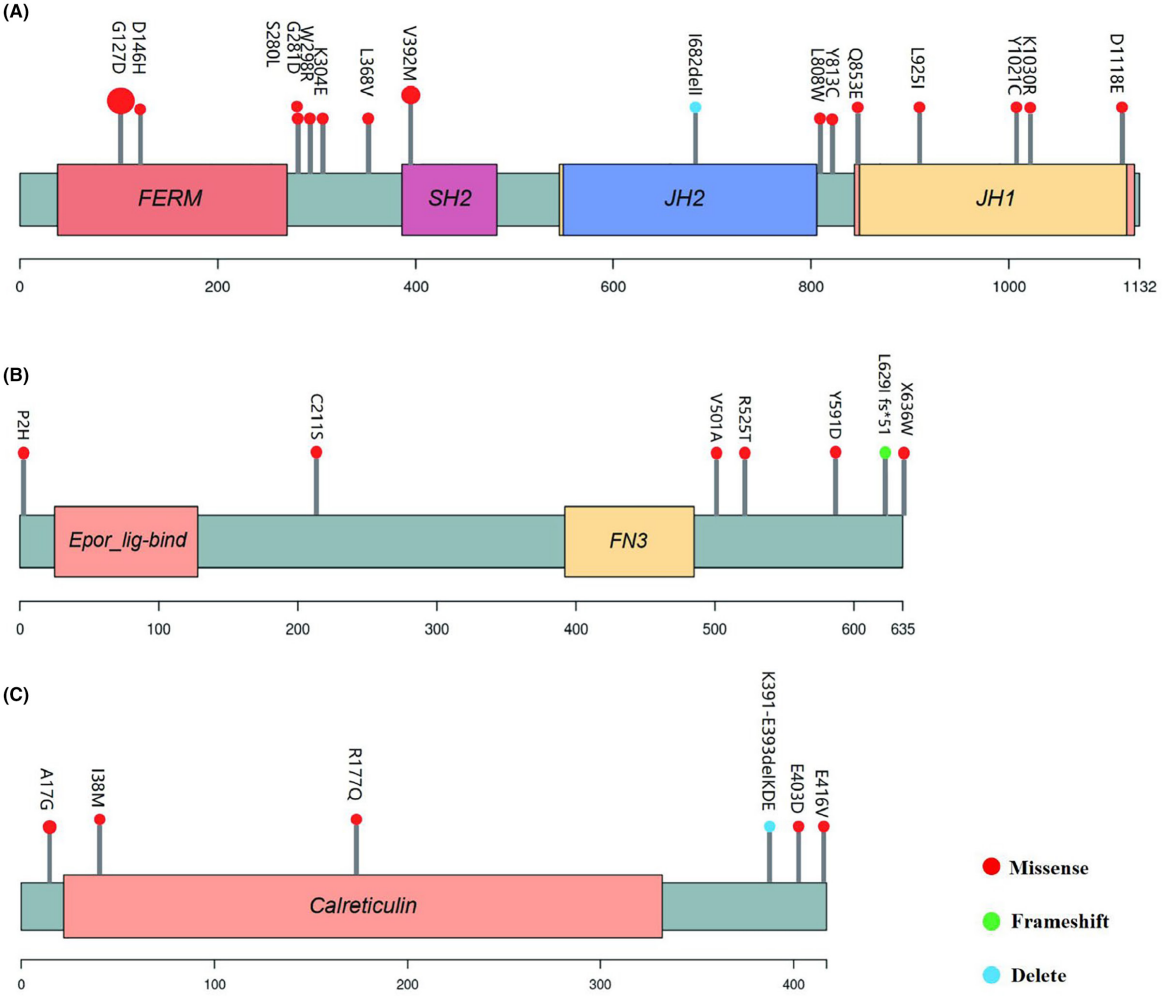
The positions of the atypical amino acid variations on the exons of driver genes, (A) *JAK2*, (B) *MPL*, and (C) *CALR*.

**FIGURE 3:**
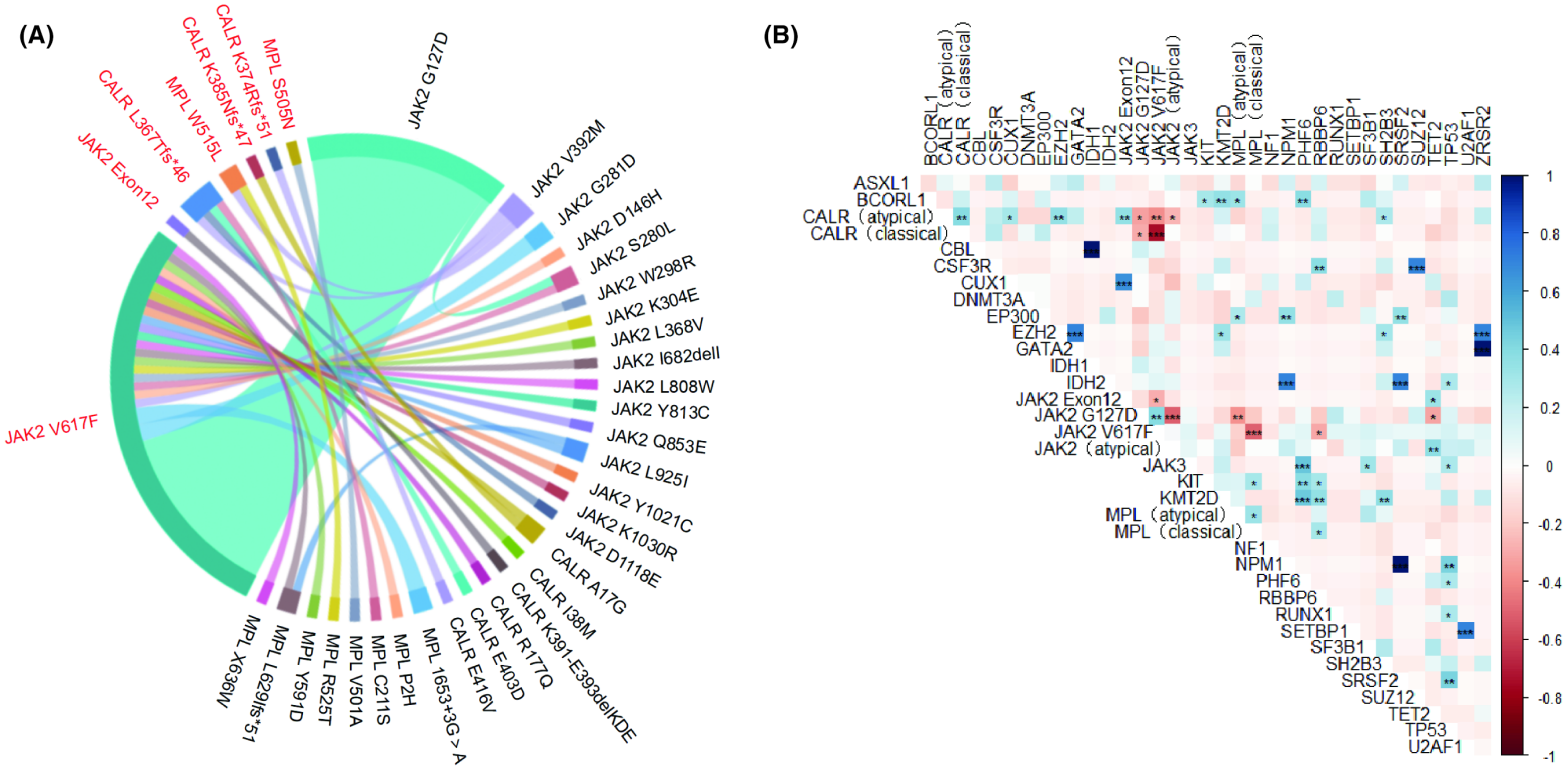
The co‐existence relationship between mutant genes. (A) Chord diagram of classical mutations and atypical variants in the driver genes *JAK2*, *MPL*, and *CALR*. (B) The relationship between gene mutations in 55 *Ph*‐negative MPN patients with atypical variants of the driver genes is shown, displaying all mutated genes and separating *JAK2*, *MPL*, and *CALR* into classical mutations and atypical variants. *JAK2* classical mutations are further divided into *JAK2* ^*V617F*^ and *JAK2 Exon12*, and *JAK2* atypical variants are divided into *JAK2 G127D* and the other atypical *JAK2* variants. Blue and red represent significant co‐existence and mutual exclusion relationships, respectively, with the *P* value denoted by. *p < 0.05, **p < 0.01, ***p < 0.001. There is a co‐existence relationship between *JAK2 G127D* and *JAK2* ^*V617F*^ (*p* < 0.01).

In this study, a total of 30 atypical variants of *JAK2*, *MPL* and *CALR* were discovered (Table 3, Figure 2), including 16 of *JAK2*, 8 of *MPL*, and 6 of *CALR*. There were 11 atypical variants of the driver genes in PV patients: *JAK2 G127D/V392M/S280L/L808W/Y813C/Y1021C*, *MPL Intron11 c.1653 + 3G > A/P2H/Y591D*, *CALR R177Q/K391‐E393delKDE*; 8 atypical variants of the driver genes were present in ET patients: *JAK2 G127D/V392M/K1030R/D1118E*, *MPL Intron11 c.1653 + 3G > A/R525T/E403D/E416V*; 15 atypical variants of the driver genes were present in PMF patients: *JAK2 G127D/G281D/D146H/W298R/K304E/L368V/I682delI/Q853E/L925I*, MPL C211S/V501A/L629Ifs*51/X636W, *CALR A17G/I38M*. Among the atypical variants of the driver genes, *JAK2 G127D* was most common (42%, 23/55), followed by *JAK2 V392M* (5%, 3/55), *JAK2 G281D*, *JAK2 S280L*, *JAK2 L925I*, *CALR A17G*, *MPL Intron11 c.1653 + 3G > A*, *MPL L629Ifs51* (4%, 2/55), and the remaining *JAK2*, *MPL*, and *CALR* atypical variants were rare individual cases (2%, 1/55). Further analysis revealed that classical mutations and atypical variants of the driver genes *JAK2*, *MPL*, and *CALR* may not be completely mutually exclusive (Figure S2). All *JAK2 G127D* mutation were coexisted with *JAK2* ^*V617F*^ (Figure 3A), and there is a co‐existence relationship between *JAK2* ^*V617F*^ and *JAK2 G127D* (*p* < 0.01) (Figure 3B). Further analysis of *JAK2* ^*V617F*^ VAF in *Ph*‐negative MPN patients with atypical variants of driver genes and without atypical variants of driver genes, we found that *JAK2* ^*V617F*^ VAF was lower in *JAK2* ^*V617F+*^/*JAK2 G127D* ^+^ patients than in *JAK2* ^*V617F+*^/*JAK2 G127D* ^*−*^ patients (median: 30.8% vs. 50.0%, *p* < 0.05, Figure S4A).

**TABLE 3:** Location, variation VAF, and functional prediction of *JAK2*, *MPL*, and *CALR* atypical variants.

Gene	Number	Position	NT change	AA change	Variants VAF (%), median (range)	SIFT	PolyPhen2	1000G	Gnome
*JAK2*	23 (PV 10, ET 7, PMF 6)	Exon5	c.380G > A	p.G127D	50.7 (15.9–92.8)	D	B	0.015	0.0195
*JAK2*	3 (PV 1, ET 2)	Exon9	c.1174G > A	p.V392M	48.5 (6.9–50.1)	D	D	0.011	0.0083
*JAK2*	2 (PMF 2)	Exon7	c.842G > A	p.G281D	37.1 (2.6 and 71.6)	D	P	–	–
*JAK2*	1 (PMF 1)	Exon5	c.436G > C	p.D146H	2.8	D	D	–	–
*JAK2*	1 (PV 1)	Exon7	c.839C > T	p.S280L	3.5	T	D	–	0
*JAK2*	1 (PMF 1)	Exon7	c.892 T > C	p.W298R	3.7	D	D	–	–
*JAK2*	1 (PMF 1)	Exon7	c.910A > G	p.K304E	23.4	T	B	–	–
*JAK2*	1 (PMF 1)	Exon9	c.1102 T > G	p.L368V	92.7	D	B	–	–
*JAK2*	1 (PMF 1)	Exon16	c.2045‐2047delTCA	p.I682delI	8.8	‐	‐	–	–
*JAK2*	1 (PV 1)	Exon18	c.2423 T > G	p.L808W	11.2	D	D	–	0.0001
*JAK2*	1 (PV 1)	Exon19	c.2438A > G	p.Y813C	1.9	D	D	–	–
*JAK2*	1 (PMF 1)	Exon19	c.2557C > G	p.Q853E	2.1	T	B	–	–
*JAK2*	1 (PMF 1)	Exon21	c.2773C > A	p.L925I	39.6	T	P	–	–
*JAK2*	1 (PV 1)	Exon23	c.3062A > G	p.Y1021C	26.0	D	D	–	–
*JAK2*	1 (ET 1)	Exon23	c.3089A > G	p.K1030R	48.5	D	D	–	0.0002
*JAK2*	1 (ET 1)	Exon25	c.3354 T > A	p.D1118E	51.6	D	D	–	–
*MPL*	2 (PV 1, ET 1)	Intron11	c.1653 + 3G > A	–	47.8 (47.4 and 48.1)	–	–	–	–
*MPL*	1 (PV 1)	Exon1	c.5C > A	p.P2H	9.6	D	D	–	–
*MPL*	1 (PMF 1)	Exon4	c.631 T > A	p.C211S	1.5	D	D	–	–
*MPL*	1 (PMF 1)	Exon10	c.1502 T > C	p.V501A	84.0	T	B	–	–
*MPL*	1 (ET 1)	Exon11	c.1574G > C	p.R525T	42.7	D	B	–	–
*MPL*	1 (PV 1)	Exon12	c.1771 T > G	p.Y591D	1.5	D	D	–	–
*MPL*	1 (PMF 1)	Exon12	c.1885‐1891delCTAAGCT	p.L629Ifs*51	3.2	–	–	–	–
*MPL*	1 (PMF 1)	Exon12	c.1908A > G	p.X636W	47.5	–	–	–	0.0010
*CALR*	2 (PMF 2)	Exon1	c.50C > G	p.A17G	48.3 (48.1 and 48.4)	D	D	0.001	0.0008
*CALR*	1 (PMF 1)	Exon2	c.114C > G	p.I38M	50.0	D	P	–	–
*CALR*	1 (PV 1)	Exon9	c.530G > A	p.R177Q	52.6	D	D	–	0
*CALR*	1 (PV 1)	Exon9	c.1171‐1179delAAAGATGAG	p.K391‐E393delKDE	47.9	–	–	–	–
*CALR*	1 (ET 1)	Exon9	c.1209A > T	p.E403D	26.2	T	B	–	–
*CALR*	1 (ET 1)	Exon9	c.1247A > T	p.E416V	10.9	D	D	–	–

*Note*: SIFT: T represents benign variants, D represents deleterious variants; PolyPhen2: B represents benign variants; P represents possibly deleterious variants.

## 4 DISCUSSION

In this study, we evaluated the incidence, gene mutation spectrum, and clinical laboratory characteristics in *Ph*‐negative MPN patients who were classical mutations positive in driver genes and carrying atypical variants of *JAK2*, *MPL* or *CALR*. We found that 15% of the *Ph*‐negative MPN patients positive for classical mutations in driver genes carried atypical variants outside *JAK2*, *MPL*, or *CALR*, with the rate being 14% in PV, 14% in ET, and 18% in PMF. No patient carrying co‐existing classical mutations in driver genes was found in this study, which is consistent with previous studies. However, we found that classical mutations of *JAK2*, *MPL*, or *CALR* can coexist with atypical variants of *JAK2*, *MPL*, or *CALR* in *Ph*‐negative MPN patients. We discovered for the first time that the incidence of *JAK2* atypical variants is higher than that of *MPL* atypical variants, and the incidence of *MPL* atypical variants is higher than that of *CALR* atypical variants among the atypical variants of driver genes. PMF had a greater variety of atypical variants of driver genes than PV and ET.

Schulze et al.^25^ found that 15% (12/82) of *JAK2* ^*V617F*^ positive patients also carry atypical variants in *JAK2* or classical mutations/atypical variants in *MPL*, but did not discover classical mutations or atypical variants in *JAK2* or *MPL* in patients positive for the classical mutation in *CALR Exon9*. In our study, 16% (45/288) of *JAK2* ^*V617F*^ positive patients carry atypical variants in *JAK2*, *MPL*, or *CALR*. However, we found that 11% (6/56) of patients who were positive for classical mutations in *CALR Exon9* also carried atypical variants in *JAK2*, *MPL*, or *CALR*. Also, we found that 25% (3/12) of patients positive for classical mutations in *MPL Exon10* carried atypical variants in *JAK2* or *MPL*. In PV, we not only found atypical variants in *JAK2*, but also in *MPL* and *CALR*.

In Western research on atypical variants of driver genes in *Ph*‐negative MPN patients, the most common is *JAK2 R1063H*.^25^, ^26^ However, in our study *JAK2 R1063H* was not found, which is speculated to be due to differences in population genetic background. The *JAK2 R1063H* is a germline variation and is an SNP (rs41316003). *JAK2 R1063H* shows a weak induction of the JAK–STAT signal in hereditary erythrocytosis, and when *JAK2 R1063H* and *JAK2 E846D* coexist, they jointly induce a stronger JAK–STAT signal activation.^27^ In this study, a total of 30 atypical variants of the driver genes were found, most of these variants have not been reported in previous studies on *Ph*‐negative MPNs. The median VAF of these atypical variants in the 40% ~ 60% range may originate from a germline, including *JAK2 G127D*, *JAK2 V392M*, *JAK2 K1030R*, *JAK2 D1118E*, *MPL c.1653 + 3G > A*, *MPL R525T*, *MPL X636W*, *CALR A17G*, *CALR I38M*, *CALR R177Q*, *CALR p.K391‐E393deIKDE*. Due to the highest incidence of *JAK2 G127D*, we detected the patient's hair follicles to confirm that *JAK2 G127D* is a germline variation. *JAK2 G127D* has been reported in *Ph*‐negative MPN and also considered to be SNP (rs56118985), but the incidence is very low in the Western world *Ph*‐negative MPN patients.^26^, ^28^, ^29^ The COSMIC cancer database shows that this mutation site has been detected in central nervous system tumors (COSM9500826), the SIFT and Mutation Taster functional prediction databases suggest that this variant is harmful. The 1000G EAS population database shows that this variant has a proportion of 1.5% in the population, and the gnomAD exome EAS population database shows that this variant has a proportion of 1.95% in the population, but *JAK2 G127D* in our *Ph*‐negative MPN cohort incidence (6.41%, 23/359) is significantly higher than the SNP. The frequent co‐existence of *JAK2 G127D* with *JAK2* ^*V617F*^ in our patient cohort warrants attention. Gill et al.^29^ found in a study of 101 cases of MPN‐MF in Hong Kong, that 16 patients had *JAK2 G127D* mutations, the classical mutations co‐existing with *JAK2 G127D* include *JAK2* ^*V617F*^, *MPL W515L/K*, *CALR Exon9 del*, which is somewhat different from our research results. In this study, all classical mutations of the driver gene that coexist with *JAK2 G127D* are *JAK2* ^*V617F*^. We found that MPN patients with the *JAK2 G127D* variation had a lower *JAK2* ^*V617F*^ VAF than those without the *JAK2 G127D* variation. We suspect that *JAK2 G127D* and *JAK2* ^*V617F*^ may have a “synergistic effect”, and further verification is needed for how they play a role in the occurrence and progression of MPN disease. This is also our next research plan.

In *Ph*‐negative MPN patients with atypical variants of driver genes, the top 10 mutation frequency were all signal transduction (classical and atypical *JAK2*, atypical *MPL*, *SH2B3* and atypical *CALR*) and epigenetic related mutations (*TET2*, *ASXL1*, *KMT2D*, *DNMT3A* and *EP300*). We found that in *Ph*‐negative MPN, mutations in *SH2B3* and *ASXL1* were significantly higher in patients with atypical variants of driver genes than in patients without atypical variants of driver genes, this finding requires further research. According to previous literature studies, *SH2B3* and *ASXL1* mutations are associated with poor prognosis of MPN patients, such as low overall survival (OS), progression of MF and leukemia transformation.^12^, ^30^, ^31^ Benton et al.^32^ found that the co‐existence of *JAK2* ^*V617F*^ and *JAK2* atypical variants was associated with an increased risk of leukemia transformation in MF patients. Therefore, we speculate that patients with additional atypical variants of driver genes may associate with a poorer prognosis. Because the patients were not dynamically followed up, so it was not possible to conduct a prognostic analysis. Patients with driver gene atypical variants may have different disease processes, and it is necessary to observe dynamics of the follow‐up through patterns of large‐scale patients or multi‐center collaboration. Our next focus will be on the mechanism of *SH2B3* and *ASXL1* mutations in *Ph*‐negative MPN with driver gene atypical variants.

## 5 CONCLUSION

The results of this study suggest that the driver genes *JAK2*, *MPL*, and *CALR* may not be completely mutually exclusive for classical mutations and atypical variants. *Ph*‐negative MPN patients with atypical variants of driver genes have a higher mutation rate of *SH2B3* and *ASXL1*, which may indicate poor prognosis. Although most of the 30 atypical variants of driver genes found in this study are predicted to be harmful functional mutations by authoritative functional databases, the prediction results may not be consistent with the actual effect produced in the organism, thus further functional validation of these variants is needed, which is also our next work plan. Currently, although the discovery of additional atypical variants of driver genes has not affected the therapeutic strategies or disease diagnosis for MPN patients, whether the existence of double or multiple mutations of driver genes will cause differences in clinical manifestations or disease progression deserves clinical attention. How the atypical variants of driver genes play a role in the occurrence and development of MPN is still unclear, and their potential biological significance and impact on prognosis need further research.

## AUTHOR CONTRIBUTIONS

**Jie Bai:** Conceptualization (lead); funding acquisition (lead); project administration (equal); writing – review and editing (equal). **Zhanlong Wang:** Conceptualization (lead); data curation (equal); formal analysis (lead); investigation (lead); methodology (equal); project administration (equal); writing – original draft (lead). **Xin Tian:** Conceptualization (equal); data curation (equal); formal analysis (equal); investigation (equal); methodology (equal); writing – original draft (equal). **Jinyu Ma:** Conceptualization (equal); data curation (equal); formal analysis (equal); investigation (equal); writing – original draft (equal). **Yuhui Zhang:** Methodology (equal); software (equal). **Wenru Ta:** Resources (equal); software (equal); supervision (equal); validation (equal); visualization (equal). **Yifan Duan:** Methodology (equal); project administration (equal). **Fengli Li:** Formal analysis (equal). **Hong Zhang:** Formal analysis (equal); software (equal). **Long Chen:** Data curation (equal); resources (equal). **Shaobin Yang:** Data curation (equal); resources (equal). **Enbin Liu:** Data curation (equal); resources (equal). **Yani Lin:** Conceptualization (equal); data curation (equal); resources (equal). **Weiping Yuan:** Funding acquisition (equal); writing – review and editing (equal). **Kun Ru:** Methodology (equal); resources (lead); writing – review and editing (equal).

## FUNDING INFORMATION

National Key Research and Development Program of China, Grant/Award number: 2020YFE0203000; National Natural Science Foundation of China, Grant/Award number: 82170117, 82270148; CAMS Innovation Fund for Medical Sciences, Grant/Award number: 2022‐I2M‐2‐003; Municipal Education Commission Scientific Research Plan Project of Tianjin, Grant/Award number: 2022ZD072, 2022KJ253.

## CONFLICT OF INTEREST STATEMENT

The authors declare no competing interests.

### ETHICS STATEMENT

The study was approved by the Ethics Committee of the Sino‐US Diagnostics Lab, Tianjin Enterprise Key Laboratory of AI‐aided Hematopathology Diagnosis (No. 202302). A written informed consent form has been provided to all participants. This study was performed in accordance with the Declaration of Helsinki.

## Supporting information


Data S1.

## Data Availability

Data available statement:All data are available upon request.
