# Supplementary material for: Clinical laboratory characteristics and gene mutation spectrum of Ph‐negative MPN patients with atypical variants of JAK2, MPL, or CALR
Source: Cancer Med. 2024 Apr 15;13(7):e7123. doi: 10.1002/cam4.7123 (PMC11017299; doi:10.1002/cam4.7123)
Supplement: Supplementary file 1 — Data S1. [file CAM4-13-e7123-s001.docx]

**Supplementary Information Data**

**Supplementary Table 1 The driver genes VAF of classical mutations and atypical variants** **in 55 *Ph*-negative MPN patients with atypical variants of driver genes**

| **Patient ID** | **Diagnosis** | **Gene** | **Position** | **NT change** | **AA change** | **VAF（%）** |
| --- | --- | --- | --- | --- | --- | --- |
| 1 | PV | JAK2 | Exon14 | c.1849G>T | p.V617F | 64.1 |
|  |  | JAK2 | Exon5 | c.380G>A | p.G127D | 15.9 |
| 2 | PV | JAK2 | Exon14 | c.1849G>T | p.V617F | 54.7 |
|  |  | JAK2 | Exon5 | c.380G>A | p.G127D | 72.2 |
| 3 | PV | JAK2 | Exon14 | c.1849G>T | p.V617F | 69.2 |
|  |  | JAK2 | Exon5 | c.380G>A | p.G127D | 81.3 |
| 4 | PV | JAK2 | Exon14 | c.1849G>T | p.V617F | 75.9 |
|  |  | JAK2 | Exon5 | c.380G>A | p.G127D | 86.7 |
| 5 | PV | JAK2 | Exon14 | c.1849G>T | p.V617F | 78.6 |
|  |  | JAK2 | Exon5 | c.380G>A | p.G127D | 87.2 |
|  |  | JAK2 | Exon7 | c.839C>T | p.S280L | 3.5 |
| 6 | PV | JAK2 | Exon14 | c.1849G>T | p.V617F | 73.8 |
|  |  | JAK2 | Exon5 | c.3800G>A | p.G127D | 82.7 |
| 7 | PV | JAK2 | Exon14 | c.1849G>T | p.V617F | 30.8 |
|  |  | JAK2 | Exon5 | c.380G>A | p.G127D | 62.4 |
| 8 | PV | JAK2 | Exon14 | c.1849G>T | p.V617F | 12.3 |
|  |  | JAK2 | Exon5 | c.380G>A | p.G127D | 47.4 |
| 9 | PV | JAK2 | Exon14 | c.1849G>T | p.V617F | 11.8 |
|  |  | JAK2 | Exon5 | C.380G>A | p.G127D | 52.1 |
| 10 | PV | JAK2 | Exon14 | c.1849G>T | p.V617F | 44.0 |
|  |  | JAK2 | Exon5 | C.380G>A | p.G127D | 27.2 |
| 11 | PV | JAK2 | Exon14 | c.1849G>T | p.V617F | 41.9 |
|  |  | MPL | Intron11 | c.1653+3G>A | - | 47.4 |
| 12 | PV | JAK2 | Exon14 | c.1849G>T | p.V617F | 52.9 |
|  |  | MPL | Exon1 | c.5C>A | p.P2H | 9.6 |
| 13 | PV | JAK2 | Exon14 | c.1849G>T | p.V617F | 78.3 |
|  |  | JAK2 | Exon19 | c.2438A>G | p.Y813C | 1.9 |
| 14 | PV | JAK2 | Exon14 | c.1849G>T | p.V617F | 73.9 |
|  |  | JAK2 | Exon18 | c.2423T>G | p.L808W | 11.2 |
| 15 | PV | JAK2 | Exon14 | c.1849G>T | p.V617F | 56.1 |
|  |  | CALR | Exon5 | c.530G>A | p.R177Q | 52.6 |
| 16 | PV | JAK2 | Exon14 | c.1849G>T | p.V617F | 86.2 |
|  |  | JAK2 | Exon9 | c.1174G>A | p.V392M | 6.9 |
| 17 | PV | JAK2 | Exon14 | c.1849G>T | p.V617F | 78.9 |
|  |  | MPL | Exon12 | c.1771T>G | p.Y591D | 1.5 |
| 18 | PV | JAK2 | Exon12 | c.1624-1629delAATGAA | p.N542-E543delNE | 40.8 |
|  |  | CALR | Exon9 | c.1171-1179delAAAGATGAG | p.K391-E393delKDE | 47.9 |
| 19 | PV | JAK2 | Exon14 | c.1849G>T | p.V617F | 55.4 |
|  |  | JAK2 | Exon23 | c.3062A>G | p.Y1021C | 26.0 |
| 20 | ET | JAK2 | Exon14 | c.1849G>T | p.V617F | 22.9 |
|  |  | JAK2 | Exon5 | c.380G>A | p.G127D | 51.2 |
| 21 | ET | JAK2 | Exon14 | c.1849G>T | p.V617F | 34.7 |
|  |  | JAK2 | Exon5 | c.380G>A | p.G127D | 45.6 |
| 22 | ET | JAK2 | Exon14 | c.1849G>T | p.V617F | 27.1 |
|  |  | JAK2 | Exon5 | c.380G>A | p.G127D | 46.5 |
| 23 | ET | JAK2 | Exon14 | c.1849G>T | p.V617F | 22.5 |
|  |  | JAK2 | Exon5 | c.380G>A | p.G127D | 50.7 |
| 24 | ET | JAK2 | Exon14 | c.1849G>T | p.V617F | 39.3 |
|  |  | JAK2 | Exon5 | c.380G>A | p.G127D | 45.8 |
| 25 | ET | JAK2 | Exon14 | c.1849G>T | p.V617F | 10.6 |
|  |  | JAK2 | Exon5 | c.380G>A | p.G127D | 48.1 |
| 26 | ET | JAK2 | Exon14 | c.1849G>T | p.V617F | 12.1 |
|  |  | JAK2 | Exon5 | c.380G>A | p.G127D | 48.1 |
| 27 | ET | JAK2 | Exon14 | c.1849G>T | p.V617F | 48.4 |
|  |  | MPL | Intron11 | c.1653+3G>A | - | 48.1 |
| 28 | ET | JAK2 | Exon14 | c.1849G>T | p.V617F | 20.5 |
|  |  | JAK2 | Exon23 | c.3089A>G | p.K1030R | 48.5 |
| 29 | ET | CALR | Exon9 | c.1099-1150del52 | p.L367Tfs*46 | 10.1 |
|  |  | JAK2 | Exon25 | c.3354T>A | p.D1118E | 51.6 |
| 30 | ET | CALR | Exon9 | c.1121-1136del16 | p.K374Rfs*51 | 36.6 |
|  |  | CALR | Exon9 | c.1247A>T | p.E416V | 10.9 |
| 31 | ET | CALR | Exon9 | c.1099-1150del52 | p.L367Tfs*46 | 36.8 |
|  |  | JAK2 | Exon9 | c.1174G>A | p.V392M | 48.5 |
| 32 | ET | CALR | Exon9 | c.1099-1150del52 | p.L367Tfs*46 | 21.4 |
|  |  | CALR | Exon9 | c.1209A>T | p.E403D | 26.2 |
| 33 | ET | MPL | Exon10 | c.1544G>T | p.W515L | 7.0 |
|  |  | JAK2 | Exon9 | c.1174G>A | p.V392M | 50.1 |
| 34 | ET | MPL | Exon10 | c.1544G>T | p.W515L | 5.8 |
|  |  | MPL | Exon11 | C.1574G>C | p.R525T | 42.7 |
| 35 | Pre-PMF | JAK2 | Exon14 | c.1849G>T | p.V617F | 29.4 |
|  |  | JAK2 | Exon5 | c.380G>A | p.G127D | 63.2 |
| 36 | Pre-PMF | CALR | Exon9 | c.1099-1150del52 | p.L367Tfs*46 | 41.5 |
|  |  | MPL | Exon4 | c.631T>A | p.C211S | 1.5 |
| 37 | Pre-PMF | JAK2 | Exon14 | c.1849G>T | p.V617F | 94.2 |
|  |  | JAK2 | Exon5 | c.436G>C | p.D146H | 2.8 |
| 38 | Pre-PMF | JAK2 | Exon14 | c.1849G>T | p.V617F | 42.3 |
|  |  | JAK2 | Exon7 | c.892T>C | p.W298R | 3.7 |
| 39 | Pre-PMF | JAK2 | Exon14 | c.1849G>T | p.V617F | 27.6 |
|  |  | JAK2 | Exon19 | c.2557C>G | p.Q853E | 2.1 |
| 40 | Pre-PMF | JAK2 | Exon14 | c.1849G>T | p.V617F | 32.4 |
|  |  | CALR | Exon2 | c.114C>G | p.I38M | 50.0 |
| 41 | Overt-PMF | JAK2 | Exon14 | c.1849G>T | p.V617F | 22.2 |
|  |  | JAK2 | Exon5 | c.380G>A | p.G127D | 45.6 |
| 42 | Overt-PMF | JAK2 | Exon14 | c.1849G>T | p.V617F | 30.1 |
|  |  | JAK2 | Exon5 | c.380G>A | p.G127D | 48.9 |
| 43 | Overt-PMF | JAK2 | Exon14 | c.1849G>T | p.V617F | 28.5 |
|  |  | JAK2 | Exon5 | c.380G>A | p.G127D | 48.7 |
| 44 | Overt-PMF | JAK2 | Exon14 | c.1849G>T | p.V617F | 56.1 |
|  |  | JAK2 | Exon5 | c.380G>A | p.G127D | 76.7 |
| 45 | Overt-PMF | JAK2 | Exon14 | c.1849G>T | p.V617F | 92.1 |
|  |  | JAK2 | Exon5 | c.380G>A | p.G127D | 92.8 |
| 46 | Overt-PMF | JAK2 | Exon14 | c.1849G>T | p.V617F | 94.7 |
|  |  | CALR | Exon1 | c.50C>G | p.A17G | 48.1 |
| 47 | Overt-PMF | JAK2 | Exon14 | c.1849G>T | p.V617F | 23.9 |
|  |  | MPL | Exon12 | c.1908A>G | p.X636W | 47.5 |
| 48 | Overt-PMF | JAK2 | Exon14 | c.1849G>T | p.V617F | 5.5 |
|  |  | JAK2 | Exon16 | c.2045-2047delTCA | p.I682delI | 8.8 |
| 49 | Overt-PMF | JAK2 | Exon14 | c.1849G>T | p.V617F | 80.2 |
|  |  | JAK2 | Exon7 | c.842G>A | p.G281D | 71.6 |
| 50 | Overt-PMF | JAK2 | Exon14 | c.1849G>T | p.V617F | 77.9 |
|  |  | JAK2 | Exon7 | c.842G>A | p.G281D | 2.6 |
| 51 | Overt-PMF | JAK2 | Exon14 | c.1849G>T | p.V617F | 85.5 |
|  |  | JAK2 | Exon9 | c.1102T>G | p.L368V | 92.7 |
| 52 | Overt-PMF | CALR | Exon9 | c.1154-1155insTTGTC | p.K385Nfs*47 | 43.5 |
|  |  | CALR | Exon1 | c.50C>G | p.A17G | 48.4 |
| 53 | Overt-PMF | JAK2 | Exon14 | c.1849G>T | p.V617F | 72.0 |
|  |  | JAK2 | Exon7 | c.910A>G | p.K304E | 23.4 |
| 54 | Overt-PMF | JAK2 | Exon14 | c.1849G>T | p.V617F | 24.0 |
|  |  | JAK2 | Exon21 | c.2773C>A | p.L925I | 39.6 |
|  |  | MPL | Exon12 | c.1885-1891delCTAAGCT | p.L629Ifs*51 | 3.2 |
| 55 | Overt-PMF | MPL | Exon10 | c.1514-1515delinsAT | p.S505N | 84.0 |
|  |  | MPL | Exon10 | c.1502T>C | p.V501A | 84.0 |

**Supplementary Table 2 The results of chromosome karyotype in 55 *Ph*-negative MPN patients with atypical variants of the driver genes**

| **Patient ID** | **Diagnosis** | **Karotype** | **Patient ID** | **Diagnosis** | **Karotype** |
| --- | --- | --- | --- | --- | --- |
| 1 | PV | 46, XX[20] | 29 | ET | 46, XY[20] |
| 2 | PV | NA | 30 | ET | 45, X, -Y[14]/46,XY[1] |
| 3 | PV | 46, XY[20] | 31 | ET | 46, XX[20] |
| 4 | PV | 46, XY[8] | 32 | ET | 46, XX[10] |
| 5 | PV | 46, XX[20] | 33 | ET | NA |
| 6 | PV | 47, XY, +9[1]/46, XY[19] | 34 | ET | 46, XX[20] |
| 7 | PV | 46, XX[20] | 35 | Pre-PMF | 46, XX[20] |
| 8 | PV | NA | 36 | Pre-PMF | 46, XX[20] |
| 9 | PV | 46, XY[20] | 37 | Pre-PMF | NA |
| 10 | PV | 46, XY[20] | 38 | Pre-PMF | 46, XY[20] |
| 11 | PV | 46, XY[20] | 39 | Pre-PMF | 46, XY[20] |
| 12 | PV | 46, XX[20] | 40 | Pre-PMF | 46, XY[20] |
| 13 | PV | 46, XY[20] | 41 | Overt-PMF | 46, XX[20] |
| 14 | PV | 46, XX[20] | 42 | Overt-PMF | NA |
| 15 | PV | 46, XY[20] | 43 | Overt-PMF | 46, XY[3] |
| 16 | PV | 46, XX[20] | 44 | Overt-PMF | NA |
| 17 | PV | 46, XX[20] | 45 | Overt-PMF | 46, XX[20] |
| 18 | PV | 46, XX[20] | 46 | Overt-PMF | 46, XY[10] |
| 19 | PV | 46, XX[20] | 47 | Overt-PMF | 46, XX[12] |
| 20 | ET | 46, XY[6] | 48 | Overt-PMF | 46, XX[20] |
| 21 | ET | 45, X, -Y[4] | 49 | Overt-PMF | NA |
| 22 | ET | 46, XX[17] | 50 | Overt-PMF | 46, XX[20] |
| 23 | ET | 46, XY[20] | 51 | Overt-PMF | NA |
| 24 | ET | NA | 52 | Overt-PMF | 46, XX[20] |
| 25 | ET | 46, XY[20] | 53 | Overt-PMF | 46, XY[15] |
| 26 | ET | 46, XX[20] | 54 | Overt-PMF | 47, XY, +8[12]/46,XY[8] |
| 27 | ET | 46, XY[20] | 55 | Overt-PMF | NA |
| 28 | ET | 46, XX[20] |  |  |  |


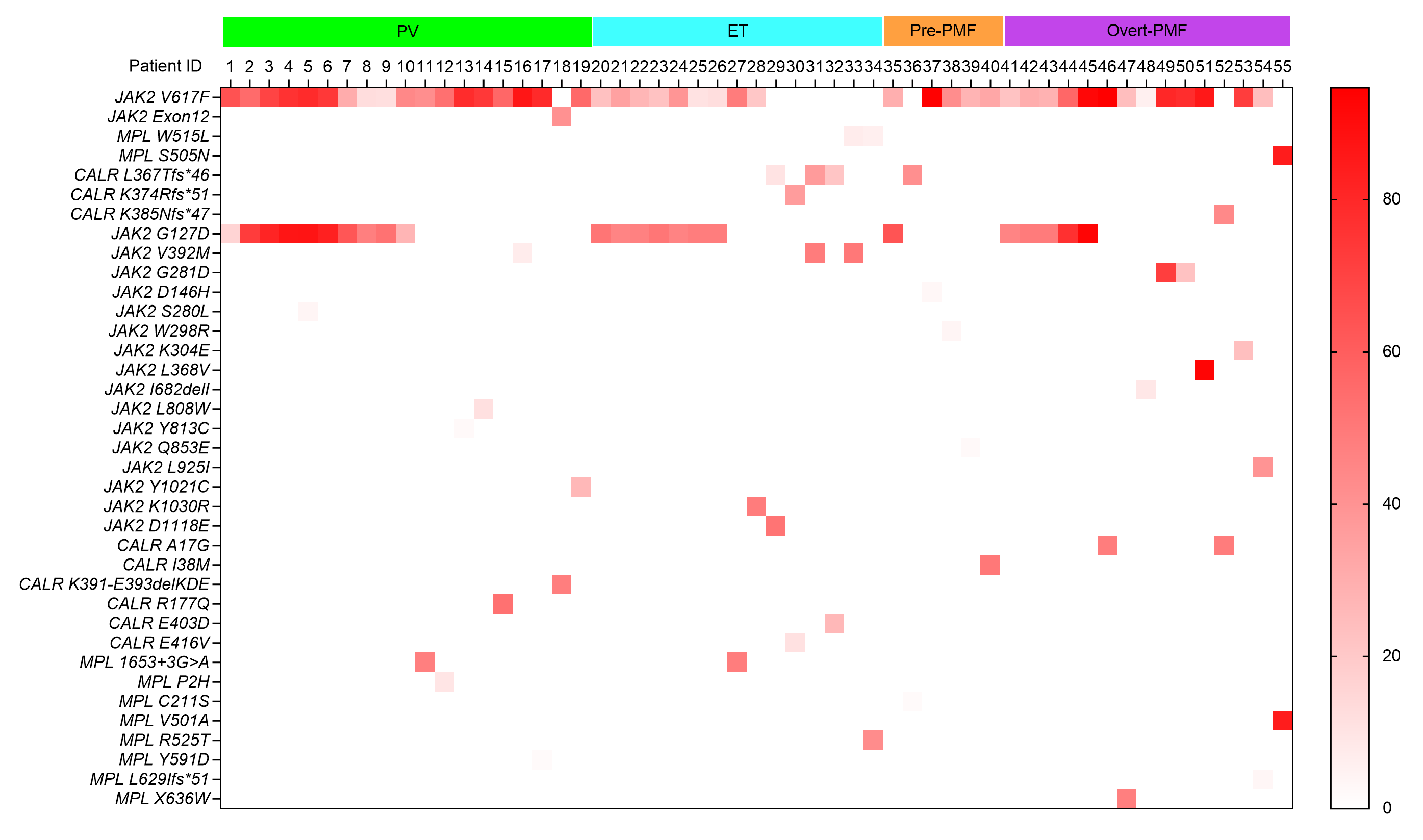


**Supplementary Figure 1 Heatmap of the co-existence of classical mutations and atypical variants in the driver genes of 55 *Ph*-negative MPN patients (color depth represents mutations VAF%).** Patients 1-19 (PV), patients 20-34 (ET), patients 35-40 (Pre-PMF), patients 41-55 (Overt-PMF).


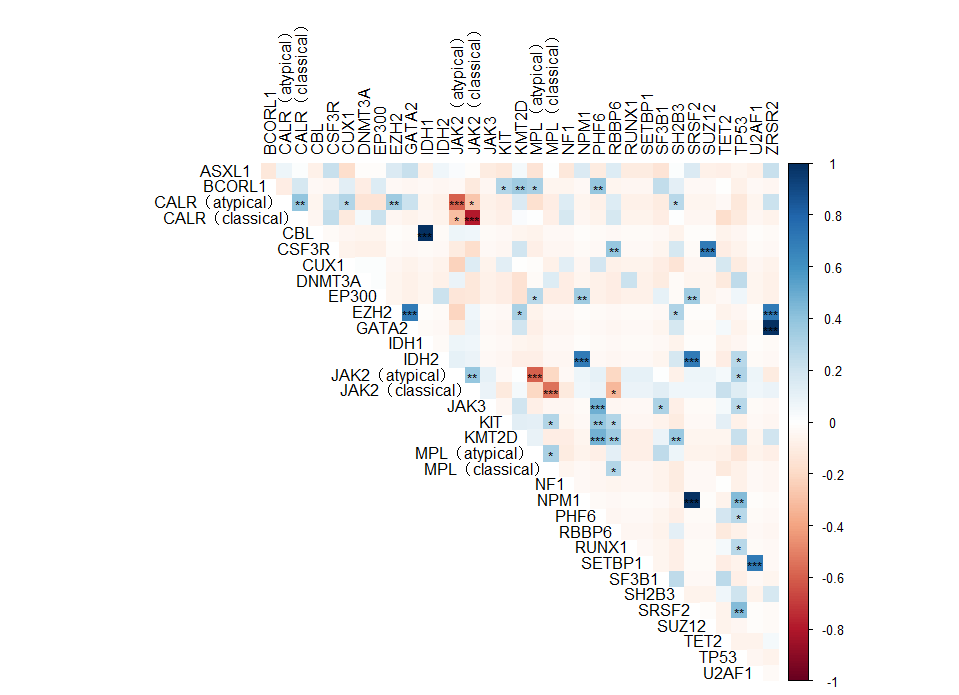


**Supplementary Figure 2** **The relationship between gene mutations in 55 *Ph*-negative MPN patients with atypical variants of the driver genes is shown, displaying all mutated genes and classifying *JAK2*, *MPL*, and *CALR* into classical mutations and atypical variants.** Blue and red represent significant co-existence and mutual exclusion relationships, respectively, with the *P* value denoted by . **P*<0.05, ***P*<0.01, ****P*<0.001. There is a co-existence relationship between *JAK2* classical mutations and *JAK2* atypical variants (*P*<0.01), *CALR* classical mutations and *CALR* atypical variants (*P*<0.01), and *MPL* classical mutations and *MPL* atypical variants (*P*<0.05). There is a mutual exclusion relationship between *JAK2* classical mutations and *MPL* classical mutations (*P*<0.001), *JAK2* classical mutations and *CALR* classical mutations (*P*<0.001), *JAK2* classical mutations and *CALR* atypical variants (*P*<0.05), *CALR* classical mutations and *JAK2* atypical variants (*P*<0.05). There is no significant co-existence or mutual exclusion relationship between *JAK2* classical mutations and *MPL* atypical variants (*P*＞0.05), *MPL* classical mutations and *JAK2* atypical variants (*P*＞0.05), *MPL* classical mutations and *CALR* atypical variants (*P*＞0.05), *CALR* classical mutations and *MPL* atypical variants (*P*＞0.05).


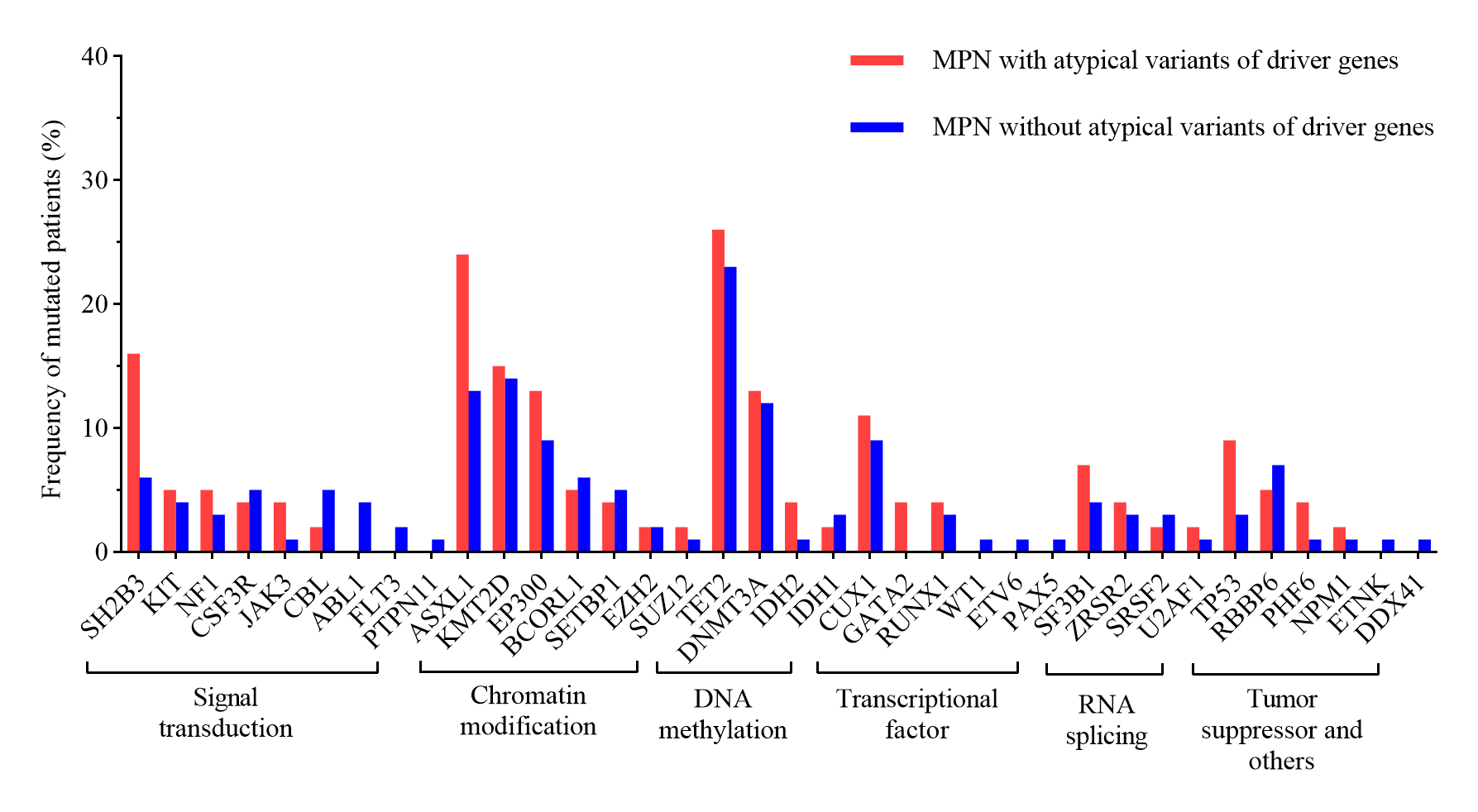


**Supplementary Figure 3** **Overview of the positive rate of mutant genes and classification of gene mutation pathways in 55 MPN patients with atypical variants of driver genes and 304 MPN patients without atypical variants of driver genes.** The histogram shows the frequency (%) of each gene mutation detected in all patients. Further analyze the occurrence rate of non-driver gene mutations in the group MPN with atypical variants of driver genes and the group MPN without atypical variants of driver genes, the incidence of *SH2B3*, *ASXL1* and TP53 mutations was higher in the MPN with atypical variants of driver genes than in the MPN without atypical variants of driver genes (*SH2B3*: 16% *vs* 6%, *P*<0.01; *ASXL1*: 24% *vs* 13%, *P*＜0.05; *TP53*: 9% *vs* 3%, *P*=0.06).


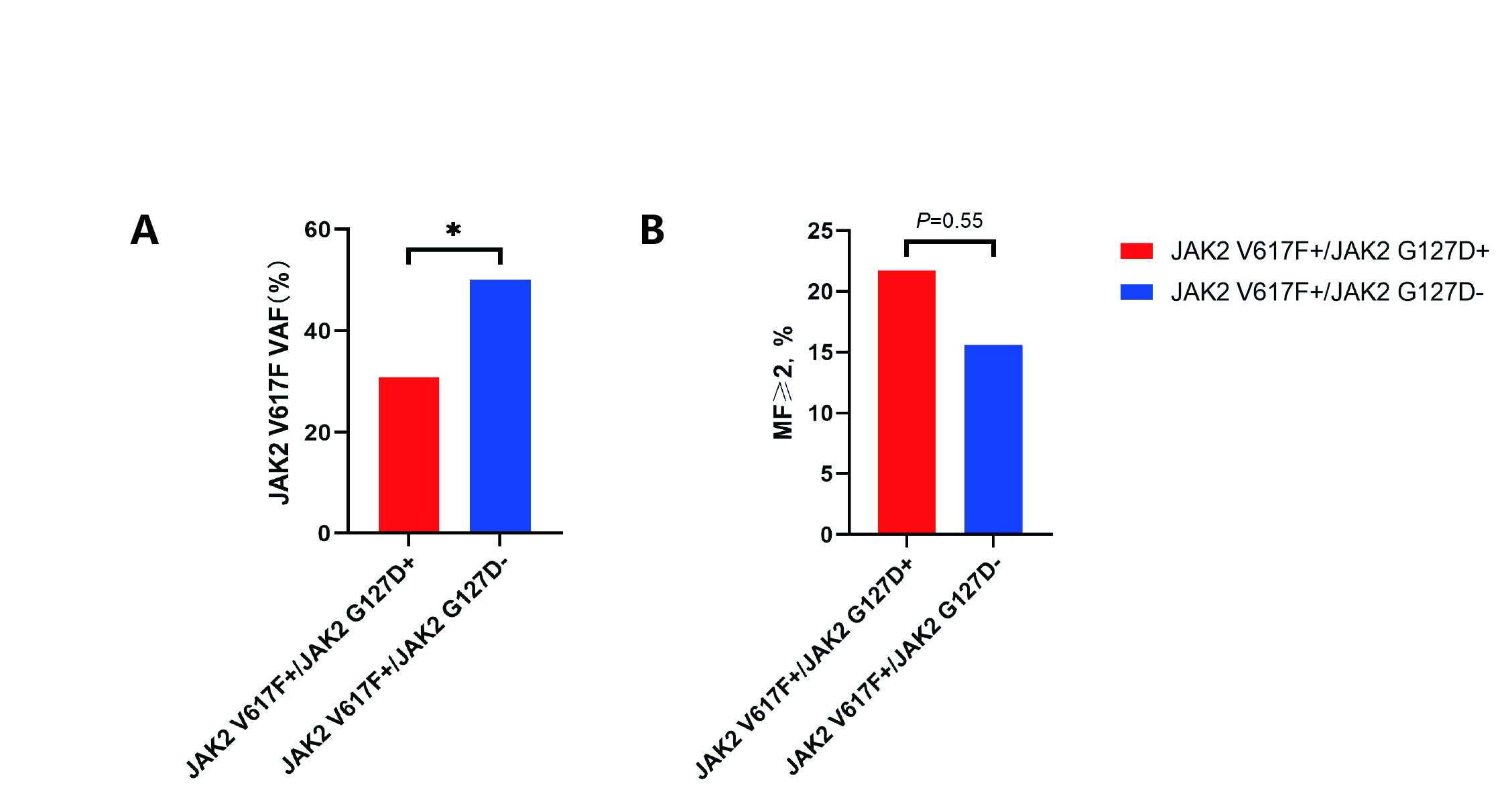


**Supplementary Figure 4 Comparison of *JAK2^V617F^* VAF (A) and the incidence of MF≥2 (B) in *JAK2^V617F^*^+^/*JAK2 G127D*^+^ MPN patients and *JAK2^V617F^*^+^/*JAK2 G127D*^-^ MPN patients.** **P*<0.05.
